# Recovery-focused self-help intervention using vodcasts for patients with personality disorder: feasibility randomised controlled trial

**DOI:** 10.1192/bjo.2023.647

**Published:** 2024-01-17

**Authors:** Youl-Ri Kim, Zhen An, Soo Wan Han, Jeong Kyung Ko, Kyung Hwa Kwag

**Affiliations:** Institute of Eating Disorders and Mental Health, Inje University, South Korea; and Department of Psychiatry, Ilsan Paik Hospital, Inje University, South Korea; Institute of Eating Disorders and Mental Health, Inje University, South Korea

**Keywords:** Personality disorders, guided self-help, randomised controlled trial, digital therapeutics, emotion regulation

## Abstract

**Background:**

Availability of long-term psychological interventions for personality disorders is limited because of their high intensity and cost. Research in evidence-based, low-intensity interventions is needed.

**Aims:**

This study aimed to examine the feasibility, acceptability and potential impact of a low-intensity, digital guided self-help (GSH) intervention that is focused on emotion regulation, recovery-oriented and provides in-the-moment delivery for patients with personality disorders.

**Method:**

We conducted a single-blind feasibility trial. A total of 43 patients with a personality disorder were recruited and randomly assigned to either a GSH arm (*n* = 22) or a treatment-as-usual arm (*n* = 21). The GSH intervention included a series of short videos offering psychoeducation and support, personalised feedback using text messages, and supportive telephone calls, for 4 weeks in addition to treatment as usual. Outcomes of emotional disturbance, emotion dysregulation, self-harm behaviours and decentring ability were measured at baseline, 4 weeks (end of intervention) and 8 weeks (follow-up).

**Results:**

All patients who attended the first session continued until the last session. There was an interaction effect between time and group on anxiety (*P* = 0.027, *Δη*^2^ = 0.10), where the GSH group showed a significant reduction in anxiety at follow-up (*P* = 0.003, *d* = 0.25). The GSH group increased in decentring ability at the end of intervention (*P* = 0.007, *d* = −0.65), and the decrease in self-harm behaviours continued until follow-up (*P* = 0.02, *d* = 0.57).

**Conclusions:**

The results suggest that a personalised digital GSH with a focus on recovery could reduce anxiety and self-harm behaviours at short-term follow-up.

Personality disorders are characterised by a long-standing, pervasive disturbance in cognition, emotional experience and expression, and patterns of behaviours that are particularly evident in interpersonal relationships.^1^ Psychoeducation programmes and cognitive–behavioural therapy have shown very limited effects in the treatment of personality disorders.^2,3^ Although previous research has demonstrated the clinical effectiveness of some long-term psychological interventions (e.g. mentalisation-based therapy and dialectical behavioural therapy) for people with some types of personality disorder, the availability is limited by their high intensity and cost.^4,5^ Although a stepped-care approach has increasingly been offered to people with personality disorders and research has been undertaken to develop low-intensity interventions,^6^ options for patients are currently limited. In a stepped-care system, low-intensity interventions are an approach to promote access to appropriate care, and only those who do not respond are provided with longer and more intensive treatment.^6^ As suggested in the previous study, low-intensity treatments may induce worries about the ability to manage difficulties without ongoing support in people with long-term conditions.^6^ To overcome this challenge, it is important to help them increase self-confidence in managing difficult situations and feelings when developing low-intensity interventions.^6^

## Key intervention components

A recovery-focused approach has been administered in mental health services.^7^ Sharing recovery experiences has been demonstrated to allow patients to gain some control over their illness^8^ and reduce symptoms in depression,^9^ substance use disorders^10^ and eating disorders.^11^ As patients with a personality disorder are resistant to treatment and, given their oscillations in mood and volition,^12^ interventions that focus on enhancing motivation to change and developing a recovery identity would be beneficial. Therefore, treatment strategies that involve sharing information and changing behaviours, accompanied by the support to develop responsibility for and trust in themselves, can be applicable to patients with personality disorders. The recovery-focused personalised approach has been suggested to have implications for borderline personality disorder (BPD),^13^ but there is a lack of research on evidence-based, recovery-focused intervention for personality disorders in general.

As emotional sensitivity in personality disorders leads to the experience of negative affect across various contexts and situations,^14^ momentary intervention provided to people as a real-time intervention in the real world^15^ may be valuable for emotion regulation. Momentary intervention strategies include visuospatial elements (visual and aural imagery) designed to interrupt the unhelpful imagery underpinning the maintenance of aversive emotional states.^16^

Based on the findings of innovative eating disorder studies that guided self-help (GSH) cognitive–behavioural therapy can be used for more severe disorders as a supplementary treatment,^17,18^ the application of GSH to personality pathology has been suggested. GSH cognitive–behavioural therapy produced treatment outcomes superior to therapist-administered cognitive–behavioural therapy or treatment as usual (TAU) alone.^19^ Traditional stepped-care models may be broadened in their methods of delivery, given that mobile technologies have been applied to self-help interventions for mental disorders. Alongside these technologies, support via telephone calls or personalised feedback are suggested to be essential, as studies that utilised such support generated larger effect sizes and had lower drop-out rates than those without such support.^20^ Thus, research on GSH utilising mobile technologies and personalised support may be of importance to provide evidence of enhanced adherence rates and intervention.

## Aims

Based on the importance of the issues addressed above, we developed a low-intensity intervention of novel skills-sharing, recovery-oriented digital GSH for patients with personality disorders. The intervention included a series of short videos that offered psychoeducation and support, and was accompanied by the guidance of a mentor to promote adherence. The aim of this study was to evaluate the feasibility and acceptability of the intervention in patients with personality disorders, when delivered in addition to usual care in out-patient settings. The primary outcome was changes in emotional disturbance and the secondary outcome was changes in self-harm behaviours.

## Method

### Trial design and participants

**Fig. 1:**
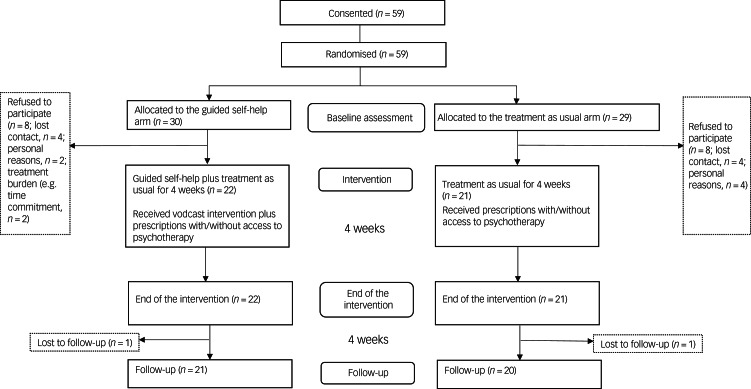
Consolidated Standards of Reporting Trials (CONSORT) flow chart.

This was a researcher-masked, parallel-group, randomised controlled trial that included a qualitative study to explore patients’ satisfaction with the intervention. An independent researcher conducted simple randomisation based on a randomised list generated in Microsoft Excel for Windows. Other researchers, mentors and participants were blind to group assignment until the completion of baseline assessment. To participate in the study, participants had to be aged 18 years or over and have a clinical diagnosis of a personality disorder based on the ICD-11 criteria.^1^ Exclusion criteria were a coexisting diagnosis of psychotic disorders and/or cognitive difficulties. The Consolidated Standards of Reporting Trials (CONSORT) flow chart in Fig. 1 shows the number of participants. In total, 59 participants were recruited between July and October 2019 (date of first recruitment: 10 July 2019) from an out-patient clinic in Seoul, South Korea. The out-patient clinic involves patients with a broad range of neurotic and mood disorder, and eating disorders. Among the recruited participants, 11 had comorbid mood disorder (19%), nine had anxiety and obsessive–compulsive disorder (15%), 20 had eating disorder (34%), five had trauma-related disorder (9%), seven had alcohol-related disorder (12%) and seven had a personality disorder only (12%). The mean duration of illness was 107.6 months (s.d. = 85.1), and the mean duration of treatment in the clinic was 39.6 months (s.d. = 47.5). Participants were randomly allocated to the GSH intervention in addition to TAU (*n* = 30) or TAU only (*n* = 29), with a 1:1 allocation ratio. Eight participants from each arm dropped out before the intervention because of personal reasons, resulting in 22 participants in the GSH arm and 21 participants in the TAU arm. As this was a feasibility study, we did not conduct a power calculation to decide the sample size.

The authors assert that all procedures contributing to this work comply with the ethical standards of the relevant national and institutional committees on human experimentation and with the Helsinki Declaration of 1975, as revised in 2008. All procedures involving human patients were approved by the Institutional Review Board of Seoul Paik Hospital of Inje University (approval number IRB-2019–02-010). Written informed consent was obtained from all participants. The study was registered with the Clinical Research Information Service (http://cris.nih.go.kr; registration number KCT0004127).

### Intervention

The skills-sharing GSH consisted of brief 2–10 min video podcasts (vodcasts) that focused on anger management, effective relationships, overcoming self-harm, emotion regulation and positive emotion induction. These were complemented by personalised feedback from a mentor on the self-monitoring forms to promote adherence. The GSH allowed the mentor and patient to determine the frequency and duration of the use of vodcasts based on patient preferences and progress. Participants were allowed to call the mentor if they had any questions on the use or contents of the vodcasts once per week (10–20 min). Table 1 describes the intervention by using the Template for Intervention Description and Replication (TIDierR) checklist.^21^

**Table 1:** Template for intervention description and replication (TIDieR) checklist

Item #	Item	Description
1.	Brief name	Recovery-focused self-help intervention using vodcasts for patients with personality disorder
2.	WHY: theoretical basis (rationale)	Evidence-based treatments for personality disorder in clinical settings are often intensive and expensive. A guided self-help intervention can complement as a low-intensity intervention in stepped care in clinical settings. The treatment strategies involving sharing information and changing behaviours via a recovery-focused approach may be applicable to patients with personality disorders, with the support to develop a responsibility for and trust in themselves
3.	WHAT: materials used in the intervention	A series of vodcasts, brief feedback to self-monitoring forms (uploaded to a secure platform) via text messages, and weekly telephone calls (tele-guidance) The vodcasts were 2–10 min video clips that covered recovery stories of people who had dealt with personality disorder in the past They addressed various strategies for reducing dysfunctional behaviours and increasing effective emotion regulation, mindful-based techniques and recovery stories The vodcasts were viewed using a portable movie player Personalised weekly feedback via text messages was provided with weekly support via brief telephone calls with a mentor
4.	WHAT: procedures used in the intervention	Participants personalised the vodcast applicability at the start of the intervention. Participants completed assessments at baseline, the end of the intervention (4 weeks) and follow-up (8 weeks)
5.	WHO: provided	Mentors were a psychiatrist and an academic psychologist
6.	HOW: modes of delivery	Vodcasts were played using a portable media player. The participants chose the vodcasts that best served their needs, and viewed each vodcast as often as they could. Mentors monitored and provided feedback on the uploaded form and supported them via weekly telephone calls. The participants underwent face-to-face assessments at baseline, the end of the intervention and follow-up
7.	WHERE: type of locations	Vodcasts were used by mobile-based technology. Mentors’ guidance was provided over text messages and telephone calls
8.	WHEN and HOW MUCH: frequency and duration	Participants viewed each vodcast as often as they could and needed. Mentors responded with brief text messages to participants’ uploaded form. Weekly telephone calls with mentors were delivered over 4 weeks for 10–20 min per call
9.	TAILORING: personalization of intervention	Mentors responded with individualized text messages to participants’ uploaded form. During weekly telephone calls, mentors and participants had together reflected on, checked, revised and supplemented the goals planned in the previous session
10.	MODIFICATION	No modification was made during the trial
11.	HOW WELL: planned fidelity to the intervention	Mentors underwent training in the delivery of text message sessions and tele-guidance
12	HOW WELL: actual fidelity to the intervention	Mentors’ adherence to the protocol for text messages and tele-guidance was regularly assessed as a matter of professional and ethical care by the lead researcher

All participants continued to receive their usual treatment, which included a brief psychotherapeutic interview and monthly or bimonthly prescriptions in an out-patient clinic. Patients in the group receiving TAU only completed assessments following the same schedule used by the GSH group. They were provided with the same vodcasts as the GSH group for 8 weeks, and feedback during their out-patient visits after the completion of the study.

#### Vodcasts

Thirty-nine vodcasts were generated by our working group of clinicians and researchers, and were played using a portable media player (PMP). The goal of the contents was to strengthen patients’ motivation to replace dysfunctional behaviours with skilled ones, and to develop the commitment to continue to use them. The vodcasts were devoted to skills-sharing complemented by recovery stories from people with lived experience. The skills-sharing focused on helping patients gain specific skills for changing behaviours related to personality problems (e.g. managing interpersonal conflicts and distressing emotions, mindfulness and positive thinking). The contents were theoretically based on two evidence-based treatments for people with personality disorders: dialectical behavioural therapy^22^ and mentalisation-based treatment.^23^

The scripts for the recovery narrative were written by clinicians based on their experiences with patients who overcame emotion dysregulation and destabilising behaviours. They were written from the perspective of a person with lived experience, reflecting the clinicians’ account on what was helpful for their patients with regard to controlling their problems, and what the patients wished they had known at the time they faced the problems. The narration was overlaid with non-distracting, soothing visual images specifically designed to help the participants focus on the message and make them more rewarding to watch. Each vodcast ended with a display of a brief prompt directly linked to the content of the vodcast to promote awareness, planning and active behaviour change.

The vodcast directory was organised as follows: (a) an introduction that focuses on support to understand personality disorders and related problems; (b) five modules on anger management, effective relationships, overcoming self-harm, emotion regulation and positive emotion induction; (c) a summary that includes exercises for reflecting on the skills learned in the previous sections and for re-evaluating the reasons for and one's confidence in changing; and (d) relaxation (see Supplementary Table 1 available at https://doi.org/10.1192/bjo.2023.647 for the list of vodcasts).

#### Potential harms

Anticipated potential harm of the vodcasts included problematic interpretations of the contents by the participants. To prevent and minimise this, the mentors answered the participants’ questions regarding the contents and corrected misinterpretations during the weekly telephone calls. Self-harming and suicidal ideation during the intervention could be another potential harm, which were assessed by an out-patient clinician. No such issue was found during the intervention.

### Study process

The GSH group had the opportunity to access a library of vodcasts. During the first face-to-face meeting, a mentor interviewed and assessed each patient's history of self-harm, emotion regulation, interpersonal problems and personality difficulties, to choose the modules needed. The patients in the GSH group were given instructions on how to use the self-help materials (vodcast modules in PMP and self-monitoring forms) and were instructed to upload the forms to a secure platform (KakaoTalk Channel for Windows, Kakao Corporation, http://pf.kakao.com/) on a weekly basis. Patients were allowed to choose vodcasts that best matched their needs, and to view each vodcast as much as they could.

Mentors responded with personalised feedback to the self-monitoring forms over Kakao Channel, and with supportive weekly telephone calls based on the protocol, if participants had any queries regarding the use of the self-help materials. The mentors were a psychiatrist and an academic psychologist trained for the guidance. Mentors’ adherence to the protocol for text messages and tele-guidance was regularly supervised with regard to professional and ethical care, by a lead researcher (Y.-R.K.).

### Measures

Assessment of participants were carried out by different researchers blind to the allocation arm. At baseline, we assessed eligibility with the Self-Report Standardized Assessment of Personality-Abbreviated Scale (SAPAS-SR),^24^ an instrument for screening patients with a personality disorder. We used a score of three or above on the Korean version of the SAPAS-SR as a cut-off point for inclusion, if a participant responded ‘yes’ to item 1 (‘In general, do you have difficulty making and keeping friends’).^25^ In 90% of the participants, pathological characteristics of personalities in the DSM-5 Section III Alternative Model of Personality Disorders^26^ were measured with the Personality Inventory for DSM-5 Short Form (PID-5-SF).^27^ Personality traits were assessed by the NEO Five-Factor Inventory (NEO-FFI).^28^ The presence of borderline pattern was screened by the Personality Disorder Questionnaire – Fourth Edition (PDQ-4+).^29^ Social functioning was assessed by the Social Functioning Questionnaire (SFQ).^30^

Participants completed the following self-reported outcome measures at baseline, 4 weeks and 8 weeks. Emotional disturbance was measured with the 21-item Depression, Anxiety and Stress Scale (DASS-21),^31^ emotion dysregulation was measured with the Difficulties in Emotion Regulation Scale (DERS),^32^ intentional self-harm was measured with the Self-Harm Inventory (SHI)^33^ and decentring (i.e. the ability to step outside of one's immediate experience and observe oneself) was measured with the Experiences Questionnaire.^34^

The GSH group was assessed by the self-monitoring form consisting of the Positive and Negative Affect Schedule (PANAS)^35^ and visual analogue scales (VAS) with two questions about the participant's positive mood and anxiety (see Supplementary Appendix 1 for detailed descriptions of each measure).

### Statistical analysis

Group differences in the demographic and clinical characteristics were analysed with an independent samples *t*-test. An analysis was conducted on attrition in the trial and non-adherence to the intervention in the GSH arm. A 2 × 3 repeated measures analysis of variance (rANOVA) was conducted to examine the main effect of and interaction between the time (baseline, end of intervention, follow-up) and group (GSH arm, TAU arm). A paired samples *t*-test compared the outcome measures between baseline and end-of-intervention and follow-up in each group. Comparisons of the self-monitoring form in the experimental group between weeks 1 and 4 were examined by a paired samples *t*-test with intention-to-treat analysis. The results of the differences are presented as the mean and effect size, if appropriate (Cohen's *d* or Hedge's *g* for paired *t*-tests, partial eta squared (*Δη*^2^) for rANOVA).^36^ As subsidiary analyses for patients with borderline pattern in the GSH arm, we made comparisons between outcomes at baseline and follow-up in patients with/without borderline pattern. Quantitative analyses were performed with SPSS 25.0 for Windows (SPSS Inc., Chicago, Illinois, USA), with a two-tailed *P*-value of 0.05. Qualitative feedback from the participants were assessed by thematic analysis.^37^

## Results

### Characteristics of the participants and their adherence to the intervention

Table 2 shows the baseline demographic and clinical characteristics of the participants. Abnormal as well as normal personality domains, personality functions, levels of depression, anxiety and stress, emotion regulation, self-harm and decentring of the participants were not statistically different between the study arms. Among the 43 participants, 23 had BPD (14 in the GSH arm and nine in the TAU arm) based on BPD screening with the PDQ-4+ (score of ≥5).^38^ There was no difference in the baseline demographic and clinical characteristics between the study participants (*n* = 43) and those who dropped out (*n* = 16), except for the NEO-FFI conscientiousness score (*t*(57) = 2.398, *P* = 0.020, *d* = 0.70). Individuals who dropped out of the study had a lower conscientiousness score (mean 36.25, s.d. = 8.21) compared with study participants (mean 41.56, s.d. = 7.31). The mean score of the Korean version of the SFQ, which assessed the participants’ levels of social dysfunction, was 10.97 (s.d. = 4.58).^30^

**Table 2:** Demographic and clinical characteristics of participants in a randomised controlled study for patients with personality disorder

	Guided self-help arm (*n* = 21)	Treatment-as-usual arm (*n* = 20)	Statistics		
	Mean (s.d.)	Mean (s.d.)	*t* or *χ^2^* (d.f.)	*P-*values	*d*
Gender					
Female	17 (80.9%)	14 (70%)	0.666 (39)	0.414	
Male	4 (19.1%)	6 (30%)			
Age, years	36.24 (10.11)	33.35 (10.54)	0.896 (39)	0.376	0.28
SAPAS-SR	5.14 (2.17)	4.15 (2.32)	1.413 (39)	0.165	0.44
SFQ	12.05 (4.89)	9.30 (4.45)	1.878 (39)	0.068	0.59
PDQ-4+, total	43.90 (17.28)	37.45 (15.95)	1.241 (39)	0.222	0.39
Borderline pattern	4.67 (2.44)	3.90 (2.43)	1.009 (39)	0.319	0.32
PID-5-SF, total	128.90 (38.60)	110.05 (33.64)	1.664 (39)	0.104	0.52
Negative affectivity	41.19 (15.20)	31.20 (15.06)	2.113 (39)	0.041	0.66
Detachment	27.57 (8.97)	22.35 (6.43)	2.133 (39)	0.039	0.67
Antagonism	16.43 (9.34)	17.80 (8.22)	−0.498 (39)	0.621	0.16
Disinhibition	22.52 (9.27)	19.40 (8.10)	1.147 (39)	0.258	0.36
Psychoticism	15.62 (8.96)	13.90 (6.13)	0.714 (39)	0.480	0.22
NEO-FFI					
Neuroticism	44.10 (10.18)	39.50 (11.44)	0.767 (39)	0.446	0.42
Extraversion	30.29 (8.17)	35.25 (10.03)	−1.514 (39)	0.136	0.54
Openness	39.76 (8.80)	41.25 (7.48)	−0.582 (39)	0.564	0.18
Agreeableness	42.48 (6.23)	44.95 (5.64)	−1.330 (39)	0.191	0.42
Conscientiousness	40.05 (7.37)	43.10 (7.45)	−1.319 (39)	0.195	0.41
DASS-21, total	28.10 (14.55)	22.60 (14.90)	1.195 (39)	0.239	0.37
Depression	9.14 (5.60)	7.80 (6.00)	0.742 (39)	0.463	0.23
Anxiety	8.48 (5.40)	5.50 (4.15)	1.972 (39)	0.056	0.62
Stress	10.48 (5.44)	9.30 (5.76)	0.673 (39)	0.505	0.21
DERS, total	100.24 (29.82)	97.15 (29.24)	0.335 (39)	0.740	0.10
Non-acceptance	13.71 (6.75)	13.25 (6.50)	0.224 (39)	0.824	0.07
Goals	16.62 (5.73)	16.55 (5.38)	0.040 (39)	0.969	0.01
Impulse	18.81 (6.92)	16.10 (7.26)	1.224 (39)	0.228	0.38
Awareness	17.10 (6.30)	18.35 (4.67)	−0.721 (39)	0.475	0.23
Strategies	22.33 (8.20)	21.55 (9.17)	0.289 (39)	0.774	0.09
Clarity	11.67 (5.69)	11.35 (5.25)	0.185 (39)	0.854	0.06
SHI	25.40 (5.94)	23.70 (3.57)	1.097 (38)	0.280	0.35
Experiences Questionnaire	27.95 (10.51)	29.20 (11.12)	−0.365 (38)	0.717	0.12

Those in the guided self-help arm were offered vodcasts intervention in addition to treatment as usual. Those in the treatment-as-usual arm were offered usual out-patient care. Analysis by independent samples *t-*test. SAPAS-SR, Self-Reported Standardized Assessment of Personality-Abbreviated Scale; SFQ, Social Functioning Questionnaire; PDQ-4+, Personality Disorder Questionnaire – Fourth Edition; PID-5-SF, Personality Inventory for DSM-5 Short Form; NEO-FFI, NEO Five-Factor Inventory; DASS-21, 21-item Depression, Anxiety, and Stress Scale; DERS, Difficulties in Emotion Regulation Scale; SHI, Self-Harm Inventory.

### Feasibility and acceptability

All of the participants who were assigned the assessment completed the assessment at the end of intervention. In the GSH group, the self-help vodcasts were watched for 38 ± 25 min per day and 21 ± 15 times per week. Participants in the GSH group reported that the intervention was overall useful and satisfying. In the thematic analysis of participant feedback on the intervention, the reasons for satisfaction were that the intervention induced positive affect (36%), provided motivational contents (23%), was easy to apply (23%) and was useful in acquiring knowledge and skills (18%). Reasons for dissatisfaction included not being able to resonate with the contents (27%) and the device being inconvenient to carry around (9%). No patient reported discomfort during the intervention (see Supplementary Table 2 for thematic analysis of participants’ qualitative feedback on the intervention).

### Effect of the GSH intervention

The two-way group × time rANOVA identified a main effect of time on the DASS-21 total score (*F*(1,39) = 4.60, *P* = 0.025, *Δη*^2^ = 0.11), depression (*F*(1,39) = 3.58, *P* = 0.043, *Δη*^2^ = 0.08), anxiety (*F*(1,39) = 3.59, *P* = 0.046, *Δη*^2^ = 0.08), stress (*F*(1,39) = 3.59, *P* = 0.045, *Δη*^2^ = 0.08), self-harm behaviour (*F*(1,36) = 4.42, *P* = 0.021, *Δη*^2^ = 0.11), and decentring (*F*(1,36) = 3.32, *P* = 0.042, *Δη*^2^ = 0.09) scores. There was a group × time interaction effect on anxiety scores (*F*(1,39) = 4.31, *P* = 0.027, *Δη*^2^ = 0.10). The effect of intervention on emotional disturbance, emotion regulation, self-harm behaviours and decentring are presented in Table 3.

**Table 3:** Efficacy outcomes measured on mood, emotion regulation, self-harm behaviours and decentring in patients with personality disorder

	Guided self-help arm (*n* = 21)			Treatment-as-usual arm (*n* = 20)			Statistics								
	Baseline	End of intervention	Follow-up	Baseline	End of intervention	Follow-up	Time (d.f. = 1.39)			Group (d.f. = 1.39)			Time×group (d.f. = 1.39)		
	Mean (s.d.)			Mean (s.d.)			*F*	*P-*value	*d*	*F*	*P-*value	*d*	*F*	*P-*value	*d*
DASS-21, total	28.10 (14.55)	19.24 (11.54)	18.67 (12.83)	22.60 (14.90)	21.75 (10.74)	20.85 (14.72)	4.60	0.025*	0.11	0.006	0.939	0.00	2.56	0.103	0.06
Depression	9.14 (5.60)	6.05 (4.13)	6.43 (4.99)	7.80 (6.00)	7.35 (3.86)	6.40 (4.83)	3.584	0.043*	0.08	0.000	0.985	0.00	1.262	0.285	0.03
Anxiety	8.48 (5.40)	5.95 (4.52)	4.76 (4.84)	5.50 (4.15)	5.50 (3.91)	5.70 (4.97)	3.592	0.046*	0.08	0.454	0.504	0.01	4.307	0.027*	0.10
Stress	10.48 (5.44)	7.24 (4.21)	7.48 (4.53)	9.39 (5.76)	8.90 (4.97)	8.75 (6.60)	3.590	0.045*	0.08	0.177	0.676	0.01	1.974	0.146	0.05
DERS, total	100.24 (29.82)	96.05 (25.49)	93.14 (27.95)	97.15 (29.24)	97.05 (26.82)	93.45 (29.44)	1.586	0.211	0.04	0.005	0.942	0.00	0.257	0.774	0.01
Non-acceptance	13.71 (6.75)	13.67 (6.37)	12.86 (5.08)	13.25 (6.50)	14.45 (6.85)	14.50 (7.69)	0.237	0.789	0.01	0.131	0.719	0.00	0.776	0.464	0.02
Goals	16.62 (5.73)	16.10 (4.78)	15.00 (6.16)	16.55 (5.38)	15.90 (4.76)	15.20 (5.05)	2.633	0.091	0.06	0.000	0.989	0.00	0.048	0.923	0.00
Impulse	18.81 (6.91)	16.71 (6.66)	16.95 (7.17)	16.10 (7.26)	16.35 (6.95)	16.00 (6.29)	1.116	0.324	0.03	0.461	0.501	0.01	1.376	0.258	0.03
Awareness	17.10 (6.30)	18.05 (5.22)	16.52 (5.59)	18.35 (4.67)	17.65 (4.69)	16.65 (6.02)	1.842	0.165	0.05	0.049	0.827	0.00	0.680	0.510	0.02
Strategies	22.33 (8.20)	19.90 (7.06)	20.33 (7.41)	21.55 (9.17)	21.30 (7.64)	20.00 (8.41)	2.373	0.110	0.06	0.002	0.968	0.00	0.917	0.390	0.02
Clarity	11.67 (5.69)	11.62 (4.76)	11.48 (4.78)	11.35 (5.25)	11.40 (4.72)	11.10 (4.45)	0.085	0.882	0.00	0.049	0.826	0.00	0.008	0.992	0.00
SHI	25.40 (5.94)	23.62 (4.10)	23.67 (4.91)	23.70 (3.57)	23.30 (3.51)	23.00 (4.04)	4.415	0.021*	0.11	0.648	0.426	0.02	2.103	0.138	0.06
Experiences Questionnaire	27.95 (10.51)	31.33 (10.61)	29.81 (11.17)	29.20 (11.12)	30.60 (9.83)	31.83 (10.84)	3.315	0.042*	0.09	0.188	0.667	0.01	1.105	0.333	0.03

Those in the guided self-help arm were offered vodcasts intervention in addition to treatment as usual. Those in the treatment-as-usual arm were offered usual out-patient care. Analysis by 2 × 3 (group×time) repeated measures analysis of variance.

DASS-21, Depression, Anxiety, and Stress Scale-21; DERS, Difficulties in Emotion Regulation Scale; SHI, Self-Harm Inventory.

*P < 0.05.

Table 4 shows the weekly progress in patients’ anxiety, mood, and positive and negative affect in the GSH arm. There was a significant reduction in the level of anxiety throughout the intervention, with a large effect size in the GSH arm (*t*(20) = 2.87, *P* = 0.009, *g* = 0.61).

**Table 4:** Weekly progress in affective symptoms throughout the guided self-help intervention in addition to treatment as usual

	Guided self-help arm (*n* = 21)				
	Week 1	Week 4	Statistics		
	Mean (s.d.)	Mean (s.d.)	*t*	*P-*value	Hedge's *g* (95% CI)
PANAS					
Positive	9.15 (6.16)	11.93 (7.38)	−1.87	0.076	−0.40 (−0.83 to 0.04)
Negative	15.55 (7.69)	12.74 (8.88)	1.46	0.160	0.31 (−0.12 to 0.74)
VAS					
Anxiety	−0.10 (2.76)	−1.24 (2.74)	2.87	0.009**	0.61 (0.15–1.07)
Positive mood	0.05 (2.33)	0.33 (2.06)	−0.47	0.641	−0.10 (−0.52 to 0.32)

Analysis by paired samples *t*-test. Intention-to-treat analysis was performed because of incomplete PANAS and VAS data on six participants. Within-group effect sizes were calculated with Hedge's *g* and 95% confidence intervals. VAS anxiety and positive mood were measured on a scale ranging from −5 to 5, with ‘−5’ indicating ‘not at all anxious/positive’ and ‘5’ indicating ‘very anxious/positive’. PANAS, Positive and Negative Affect Scale; VAS, visual analogue scale.

**P < 0.01.

We then conducted *post hoc* comparisons between baseline and end of intervention, and baseline and follow-up in each group (see Supplementary Table 3 for comparison of outcomes between baseline and end of intervention or follow-up in the GSH arm and TAU arm). In the GSH arm, the levels of depression, anxiety and stress were decreased at the end of intervention (depression: *t*(20) = 2.41, *P* = 0.026, *d* = 0.59; anxiety: *t*(20) = 2.38, *P* = 0.028, *d* = 0.52; stress: *t*(20) = 2.56, *P* = 0.019, *d* = 0.56), among which the reductions in anxiety and stress remained at follow-up (anxiety: *t*(20) = 3.33, *P* = 0.003, *d* = 0.25; stress: *t*(20) = 2.46, *P* = 0.023, *d* = 0.6). Decentring ability was improved at the end of intervention (*t*(20) = −2.98, *P* = 0.007, *d* = −0.65), whereas self-harm behaviours evaluated by the SHI reduced continuously up to the period of follow-up compared with baseline (*t*(19) = 2.55, *P* = 0.02, *d* = 0.57). In the TAU group, there was no difference between either baseline and end of intervention or baseline and follow-up in the outcome variables.

## Discussion

### Main findings

The present study demonstrates that a randomised controlled trial of GSH, which is focused on emotion regulation, recovery-oriented and provides in-the-moment delivery, is feasible and potentially effective for patients with personality disorders. The intervention included a series of short videos providing educational materials on regulating emotion and/or improving mood, which were accompanied by text messages and telephone calls to promote adherence and provide support. The results of our study indicated good acceptance and adherence to the intervention. The usage of the self-help vodcasts was higher in patients with personality disorders in this study compared with the usage in previous eating disorders studies.^39,40^ The use of the self-help videos varied across the participants. An interaction effect between time and group on reduced anxiety symptoms was found. Participants in the active arm of the trial reported significant improvements in decentring abilities and self-harm behaviours.

The high adherence observed in the study might be partly attributed to the minimal but continuous feedback the participants received from their mentors. Participants discussed their progress and questions during the 10–20 min weekly telephone calls with the mentors. This might have motivated them to complete the intervention via the rapport built with their mentors. Further studies on the role of the patient–mentor interaction in adherence rate might provide insights into the optimal frequency and form (i.e. text message, telephone call and in-person visit) of the interaction.

The quantitative data collected from the participants demonstrated a significant effect of the GSH on anxiety, but there was no significant effect on emotion dysregulation. These findings align with other studies involving patients with eating disorders, in which GSH intervention produced group differences in reduced anxiety symptoms, but not in disorder-specific symptoms.^39,41^ Recent findings suggest an alternative perspective that recovery narratives may not induce direct changes, thus highlighting the need for assessments of the underlying mechanisms of recovery narratives.^42^

A meaningful finding is that participants in the active arm of the trial reported a significant preceding improvement in positive affect, whereas self-harm behaviours decreased progressively to follow-up. These results are consistent with the idea that self-harm is maintained by negative reinforcement in the form of the avoidance of negative emotion.^43^ The findings also suggest that exposure to recovery narratives may not directly decrease self-harm, but may promote positive affect as well as the ability to endure negative responses.

The qualitative analysis of participant feedback provided additional support for the benefits that some people with a personality disorder may gain from the intervention here. Study participants valued the vodcasts for helping them cope with their interpersonal relationships and emotional difficulties, motivating them to have goals and a willingness to change, letting them acquire knowledge and skills, and making them feel positive about themselves. A few reasons for dissatisfaction were boredom caused by unengaging contents with audio repetition and the inconvenient device. Recovery narratives generated by people with lived experiences may improve the content via a sense of peer support. Also, developing more effective methods of delivery is of interest in future studies to make it less tedious and help patients stay focused and adhere to the intervention. Although the PMP did not require internet access, the device was inconvenient to handle for some participants. Using smartphone applications or web platforms might be a better alternative for increased accessibility.

### Strengths and limitations

To the best of our knowledge, this was the first randomised controlled trial to provide self-help intervention for patients with personality disorders in a non-Western country. Although the results of the study were promising overall, a few limitations need to be addressed. First, given the small sample size, the effects of the intervention need to be replicated in a larger study. Second, because of the relatively short follow-up term, it is unclear whether the effects observed in the study are enduring. In the same vein, latent harms were not measured because of the short term of the follow-up study. Follow-up studies with longer terms are needed to assess latent harms as well as sustained effects of the intervention. Third, we did not have a formal structured interview to confirm ICD-11 personality disorder diagnoses. Rather, all participants were clinically diagnosed by the psychiatrists who treated them. We do not think this process was less accurate than a structured interview, as an accurate diagnosis of a personality disorder needs to be evaluated from a longitudinal perspective. Finally, although interventions for personality disorders should focus on helping people improve their adaptability to daily life with their existing problems rather than reducing or removing personality problems,^5^ we did not collect information on social adjustment, motivation or self-efficacy.

### Clinical implications

The results suggest that the GSH intervention that is focused on emotion regulation, recovery-oriented and provides in-the-moment delivery may be an option for low intensity intervention for patients with personality disorders. In general, the results align with the findings in other low-intensity interventions for personality disorders that short-term interventions might be effective in alleviating symptoms.^44^ The current study adds to the literature with its finding of the sustained decrease in anxiety and self-harm behaviours at follow-up. Meanwhile, the intervention using recovery narratives and images is feasible and acceptable to Korean patients, suggesting that they may be the key components for overcoming cultural barriers. The intervention would be enhanced by adding contents that directly reflect patients’ experiences and by increasing accessibility through smartphone applications or web platforms. Overall, the outcomes of the current feasibility trial are supportive of larger-scale trials of vodcast interventions, with some modifications to the contents, device and methods of delivery to reflect the participants’ feedback from this study. Interaction with mentors, which is deemed to have contributed to the high adherence rate, is a key component that needs to be included and further studied to optimise its use. Clinical trials with sufficiently large samples are needed to evaluate minimum clinically significant differences between groups.

In conclusion, the recovery-focused self-help intervention using vodcasts, with minimum and personalised feedback of mentors, was feasible and preliminarily effective in decreasing anxiety and self-harm behaviours in patients with personality disorders.

## Supporting information

Kim et al. supplementary material Kim et al. supplementary material

## Data Availability

The data that support the findings of this study are available from the corresponding author, Y.-R.K., upon reasonable request. The data are not publicly available due to their containing information that could compromise the privacy of research participants.
