# Supplementary material for: Recovery-focused self-help intervention using vodcasts for patients with personality disorder: feasibility randomised controlled trial
Source: BJPsych Open. 2024 Jan 17;10(1):e31. doi: 10.1192/bjo.2023.647 (PMC10897696; doi:10.1192/bjo.2023.647)
Supplement: Kim et al. supplementary material [file S2056472423006476sup001.docx]

**Supplementary Text 1**

**Measures**

**Self-reported baseline assessment of personality disorder**

*Self-Report Standardized Assessment of Personality-Abbreviated Scale (SAPAS)*. The SAPAS-SR is a brief, self-reported instrument for screening patients with personality disorder. It consists of 8 items scored as "Yes” or “No.” The best cut-off point is 4 in both original and Korean^1^ versions. The Korean version had a good reliability, with Cronbach’s alpha ranging from 0.71 to 0.81.

*Social Functioning Questionnaire (SFQ)*. The SFQ assesses social functioning and consists of 8 items, scoring functioning in the workplace, financial status, family relationships, sexual relationships, and leisure activities. Each item is evaluated on a 4-point Likert scale (0–3), with the total scores ranging from 0 to 24 points. The Korean version of the SFQ is a reliable instrument (Cronbach’s alpha = 0.81)^2^.

*Personality Disorder Questionnaire–4+ (PDQ–4+)*. The PDQ–4+ is a self-report measure that assesses 10 specific personality disorders and 2 personality disorders proposed in the Diagnostic and Statistical Manual of Mental Disorders, Fourth Edition (DSM-IV)^3^ with 99 items in a true-false format. The Korean version of the PDQ–4+ is a reliable instrument with Cronbach’s alpha value of 0.91^4^.

*Personality Inventory for DSM-5 Short Form (PID-5-SF)*. The PID-5-SF is a self-reported inventory with a 4-point response scale (0 = very false or often false to 3 = very true or often true). It was developed to evaluate the pathological characteristics of patient personalities in DSM-5 Section III AMPD. It contains 25 lower-order facet scales of 25 personality facets of personality disorders. The PID-5-SF was developed with 100 items with the same facets and the same factor structure as the PID-5. The Korean version of the PID-5-SF^5^ was used in this study, in which the factor structures were identical to those of the PID-5. Cronbach’s alpha value of the Korean version of the PID-5-SF facet scales in the community sample ranged from 0.63 (Irresponsibility facet) to 0.89 (Anxiousness facet), with a median alpha of 0.77.

*Neuroticism-Extraversion-Openness Five-Factor Inventory (NEO-FFI)*. The NEO-FFI is a 60-item self-reported measure of 5 personality factors. The 5 subscales consist of neuroticism, extraversion, openness, agreeableness, and conscientiousness. The Korean version of the NEO-FFI was validated by Lee^6^.

**Self-reported clinical measures at baseline, 4 weeks, and 8 weeks**

*Depression Anxiety Stress Scale (DASS)*. The DASS-21 is a 21-item, 3-scale self-reported measure of depression, anxiety, and stress. Higher scores are related to higher levels of depression, anxiety, and stress. The Korean scale has been validated and found to have good reliability, with Cronbach’s alpha scores of 0.87 for depression, and 0.83 for anxiety and stress^7^.

*Difficulties in Emotion Regulation Scale (DERS)*. The DERS is a 36-item self-reported instrument measuring emotion dysregulation. It consists of 6 subscales: awareness, clarity, goals, impulse, non-acceptance, and strategies. The Korean version of the DERS is a reliable and valid instrument^8^. In this study, Cronbach’s alpha value for the total score was 0.95.

*Self-Harm Inventory (SHI).* The SHI assesses patients’ intentional self-harm via 22 items, with higher scores indicating greater severity of self-harm behaviors. The Korean version of the SHI was validated by Kim et al^9^.

*Experiences Questionnaire (EQ)*. The EQ is a 20-item self-reported instrument measuring decentering (i.e., the ability to step outside of one’s immediate experience and observe oneself, which has been discussed as a common therapeutic mechanism in many psychotherapeutic theories).

**Weekly self-reported measures after the use of vodcasts**

*Positive Affect and Negative Affect Schedule (PANAS).* The PANAS is a 20-item self-reported measure that consists of 10-item scales for positive and negative affect, respectively. The Korean version of the PANAS was shown to be reliable^10^.

*Visual Analog Scale (VAS).* The VAS had 2 questions asking about the participant’s positive mood (“Are you feeling good?”) and anxiety state (“Are you anxious?”) on a scale from -5 to +5 (-5 indicated ‘very unlikely to be feeling good’ and +5 indicated ‘very likely to be feeling good’ in the positive mood scale; -5 indicated ‘not at all anxious’ and +5 indicated ‘very anxious’ in the anxiety state scale).

**References**

1. Choi J, Hwang SJ, Pai D, Hwang ST, Kim, YR. Diagnostic efficiency of personality disorder screening tool; The Korean version of self-report standardized assessment of personality-abbreviated scale: preliminary validation study. *J Korean Neuropsychiatr Assoc* 2015; **54**: 534-41.
2. Kim YR, Hwang ST, Kim SG, Lee HS. Social function in patients with personality disorder diagnosed by single dimensional severity using Korean version of Social Functioning Questionnaire. *J Korean Neuropsychiatr Assoc* 2015; **54**: 523-33.
3. American Psychiatric Association. *Diagnostic and Statistical Manual of Mental Disorders* (4^th^ edn). APA, 1994
4. Kim DI, Choi MR, Cho EC. The preliminary study of reliability and validity on the Korean version of personality disorder questionnaire-4+(PDQ-4+). *J Korean Neuropsychiatr* Assoc 2000; 525-38.
5. Hong TH, Kim YR, Hwang, ST. Construction and validation of the Korean version of the Personality Inventory for DSM-5 Short Form (K-PID-5-SF). *Kor J Clin Psychol*, 2018; **37**: 396-410.
6. Lee K. Factor structure and maladaptive group profiles of the Revised NEO Personality Inventory for Koreans. *Doctoral dissertation,* Pusan National University, 1995.
7. Jun D, Johnston V, Kim JM, O’Leary S. Cross-cultural adaptation and validation of the Depression, Anxiety and Stress Scale-21 (DASS-21) in the Korean working population. *Work* 2018; **59**: 93-102.
8. Cho YR. Assessing emotion dysregulation: Psychometric properties of the Korean version of the Difficulties in Emotion Regulation Scale. *Kor J Clin Psychol* 2007; **26**: 1015-38.
9. Kim S, Woo S, Koo H, Lee J. Validation of the Korean version of the self-harm inventory (K-SHI). *Cognitive Behavior Therapy in Korea* 2019; **19**: 205-28.
10. Lee HH, Kim EJ, Lee MK. A validation study of Korea positive and negative affect schedule: The PANAS scales. *Kor J Cliln Psychol* 2003; **22**: 935-46.

**Supplementary Table S1. List of vodcasts used in the study**

| Module | Title |
| --- | --- |
| Introduction | Battle without a winner |
|  | Rethinking thoughts |
|  | Self-identity crisis |
| Module 1:  Anger management | Handling impulsiveness |
|  | Handling anger |
|  | The reason why you are angry |
|  | Emotional intelligence: growth through anger |
|  | Understanding your own anger patterns |
| Module 2:  Effective relationships | Trust of others |
|  | Practicing new interpersonal relationships |
|  | Building healthy relationships  Three important relationship goals  A gap between emotion and action  There exists *grey* between *black* and *white* |
| Module 3:  Overcoming self-harm | Defeating habitual self-harming behaviors  A sense of relief from understanding self-harm |
|  | Group therapy for self-harming behaviors  Pharmacotherapy for self-harming behaviors |
|  | Dealing with the impulse to self-harm |
|  | Asking for help |
|  | Managing self-harming behaviors |
| Module 4:  Emotion regulation | Adapting to the tide of emotions  How to regulate emotions  Accepting oneself |
|  | About shame |
|  | Understanding emotions |
|  | About mindfulness |
|  | Enduring and handling stress |
| Module 5:  Positive emotion induction | The role of emotion  Effective emotion regulation  Focusing on the bright side  Becoming merciful  Improving self-esteem |
| Mindfulness | Respiration  Emotion  Relaxation |
| Summary | The hope named *recovery* |
|  | From silence to happiness |
|  | The road to recovery: compassion |

**Supplementary Table S2.** **Thematic analysis of participants’ qualitative feedback on the intervention**

| *Theme Examples* | |
| --- | --- |
| **What was your reason for satisfaction with the vodcast?** | |
| Applicability | -I was able to apply the vodcast content to interpersonal relationships in work settings.  -I reflected on the vodcast content when I was experiencing difficulties in working with classmates.  -The vodcast content helped me when I was struggling with my emotions.  -I’ll try to find what I’m good at, and improve my self-esteem.  -I want to and will love myself.  -I won’t give up on myself.  -It’s important to express my emotions and opinions in an appropriate manner, and I’ll try to do so from now on.  -I felt confident on my way to work.  -I felt calm.  -I could be in a positive mood.  -I felt comfortable.  -I felt like I was being comforted by someone.  -I learned how my brain reacts to threatening emotional signals through psychoeducation.  -I learned how to calm myself when I become too emotional and was tempted to end a relationship impulsively.  -I learned how to focus on the present instead of being obsessed with my past or future. |
| Motivational contents |  |
| Positive affect |  |
| Acquisition of knowledge and skills |  |
| **What was your reason for dissatisfaction with the vodcast?** | |
| Confrontational contents | -The content just felt like a mere justification of an adverse situation that one is responsible for.  -I couldn’t relate to the content as it was different from the situation I’m currently in.  -I couldn’t agree with the content as it contradicted my experience.  -It’s repetitive and boring, though the content itself is good.  -The running time of the meditation sessions was too short.  -The device (portable media player) was inconvenient to use. |
| Unengaging content |  |
| Inconvenience |  |

|  | **Treatment as usual + vodcast self-help intervention (N=21)** | | | | | | **Treatment as usual (N=20)** | | | | | | |
| --- | --- | --- | --- | --- | --- | --- | --- | --- | --- | --- | --- | --- | --- |
|  | **Baseline vs End of Intervention** | | | **Baseline vs Follow-up** | | | **Baseline vs End of Intervention** | | | **Baseline vs Follow-up** | | | |
|  | ***t(df)*** | ***p-value*** | ***d*** | ***t(df)*** | ***p-value*** | ***d*** | ***t(df)*** | ***p-value*** | ***d*** | ***t(df)*** | ***p-value*** | ***d*** |  |
| DASS-21, total | 2.69(20) | 0.014^*^ | 0.59 | 2.81(20) | 0.^.^011^*^ | 0.69 | 0.51(20) | 0.613 | 0.09 | 0.48(19) | 0.638 | 0.12 |  |
| Depression | 2.41(20) | 0.026^*^ | 0.59 | 1.86(20) | 0.077 | 0.51 | 0.49(20) | 0.631 | 0.10 | 1.04(19) | 0.313 | 0.26 |  |
| Anxiety | 2.38(20) | 0.028^*^ | 0.52 | 3.33(20) | 0.003^**^ | 0.25 | 0.00(20) | 1.000 | 0.00 | -0.17(19) | 0.868 | -0.04 |  |
| Stress | 2.56(20) | 0.019^*^ | 0.56 | 2.46(20) | 0.023^*^ | 0.60 | 0.77(20) | 0.448 | 0.13 | 0.38(19) | 0.706 | 0.09 |  |
| DERS, total | 0.85(20) | 0.408 | 0.19 | 1.17(20) | 0.257 | 0.25 | 0.33(20) | 0.748 | 0.04 | 0.93(19) | 0.364 | 0.13 |  |
| Non-acceptance | 0.04(20) | 0.971 | 0.01 | 0.60(20) | 0.555 | 0.14 | -0.79(20) | 0.439 | -0.14 | -1.02(19) | 0.318 | -0.18 |  |
| Goals | 0.55(20) | 0.590 | 0.12 | 1.29(20) | 0.214 | 0.27 | 0.92(20) | 0.370 | 0.15 | 1.47(19) | 0.159 | 0.26 |  |
| Impulse | 1.82(20) | 0.084 | 0.40 | 1.43(20) | 0.168 | 0.26 | -0.13(20) | 0.895 | -0.02 | 0.10(19) | 0.922 | 0.02 |  |
| Awareness | -0.79(20) | 0.441 | -0.17 | 2.08(20) | 0.051 | 0.10 | 0.75(20) | 0.465 | 0.15 | 1.91(19) | 0.071 | 0.34 |  |
| Strategies | 1.96(20) | 0.064 | 0.43 | 1.46(20) | 0.160 | 0.26 | 0.51(20) | 0.619 | 0.07 | 1.10(19) | 0.286 | 0.18 |  |
| Clarity | 0.05(20) | 0.958 | 0.01 | 0.14(20) | 0.887 | 0.04 | 0.25(20) | 0.807 | 0.03 | 0.37(19) | 0.714 | 0.06 |  |
| SHI | 1.93(20) | 0.068 | 0.42 | 2.55(19) | 0.020^*^ | 0.57 | 1.98(20) | 0.062 | 0.16 | 0.52(17) | 0.610 | 0.09 |  |
| EQ | -2.98(20) | 0.007^**^ | -0.65 | -1.15(19) | 0.264 | -0.26 | -0.970(20) | 0.344 | -0.14 | -1.15(17) | 0.266 | -0.16 |  |
| *Note.* Analysis by paired samples *t*-test  *Abbreviations.* DASS-21, Depression, Anxiety, and Stress Scale-21; DERS, Difficulties in Emotion Regulation Scale; SHI, Self-Harm Inventory; EQ, Experiences Questionnaire.  *^*^p < 0.05, ^**^p<0.01.^***^p<0.001.* | | | | | | | | | | | | |  |

**Supplementary Table S3.** **Comparison of outcomes between baseline and the end of intervention or follow-up in the GSH arm and the TAU arm**
